# Digital training needs, cognitive learning ability, and teaching ability of rural teachers: the mediating role of digital literacy and the moderating role of learning engagement

**DOI:** 10.3389/fpsyg.2026.1821710

**Published:** 2026-05-12

**Authors:** Xinyu Kong

**Affiliations:** Institute of Higher Education, East China Normal University, Shanghai, China

**Keywords:** cognitive learning ability, digital literacy, digital training needs, learning engagement, rural teachers, teaching ability

## Abstract

**Background:**

In the context of educational digital transformation, the impact mechanism of digital training needs on teachers’ cognitive learning ability and teaching ability remains unclear.

**Objective:**

This study aims to examine how digital training needs are related to teachers’ cognitive learning ability and teaching ability, and to explore the mediating role of digital literacy and the moderating role of learning engagement.

**Methods:**

Scales for digital training needs, learning engagement, digital literacy, cognitive learning ability, and teaching ability were used. A total of 844 valid samples were collected from rural teachers in North China. SPSS, AMOS 26.0, and the PROCESS macro were employed for common method bias testing, multicollinearity testing, correlation analysis, and moderated mediation analysis.

**Results:**

Digital training needs were positively associated with cognitive learning ability and teaching ability, with moderate direct effects (β = 0.396 and β = 0.180, respectively). Digital literacy showed significant positive mediating effects in both pathways (β = 0.490 for cognitive learning ability; β = 0.722 for teaching ability). Learning engagement negatively moderated the association between digital training needs and digital literacy (β = −0.098), indicating that the positive effect weakened as learning engagement increased.

**Conclusion:**

The findings suggest that teacher training programs should be aligned with teachers’ actual digital training needs and prioritize the development of digital literacy as a key pathway to improving both learning and teaching outcomes. In addition, differentiated support may be needed for teachers with different levels of learning engagement.

## 1 Background

In the context of the ongoing digital transformation in education, digital technologies are reshaping how teachers access resources, conduct teaching, and engage in professional development (Wang et al., 2024). However, the benefits of this transformation are not equally distributed across regions (Zhao et al., 2021). Compared to urban schools, rural schools still face multiple limitations regarding technological infrastructure, digital resource availability, and training opportunities, resulting in rural teachers being more likely to encounter a series of challenges, such as “insufficient capacity—limited access to resources—difficulties in application transformation in digital teaching contexts (Zhao, 2024). Consequently, the overall improvement in digital literacy is slower. Due to factors such as educational background, inadequate technological infrastructure, and limited training opportunities, many rural teachers possess relatively low levels of digital literacy (Li, 2025). As digital technologies become more widespread, rural teachers’ demand for digital training has increased substantially. This trend makes it important not only to document the existence of training needs, but also to explain how such needs may translate into teachers’ learning and teaching development under constrained rural conditions.

However, existing research and practice have focused more on what training provision offers than on the mechanisms through which teachers’ training needs are translated into meaningful capability development (Patfield et al., 2023; Popova et al., 2022). From the perspective of human capital theory, training can be understood as an investment in teachers’ knowledge and skills, but its effectiveness depends on whether such investment is aligned with teachers’ actual developmental needs and can be transformed into productive professional competence (Becker, 1964). At the same time, from a self-regulated learning perspective, teachers are not passive recipients of training; rather, their learning outcomes depend on how they mobilize motivation, attention, strategy use, and sustained engagement during the learning process (Zimmerman, 2002). These two perspectives together suggest that digital training needs should not be treated merely as a descriptive indicator of demand, but as a developmental signal whose effects may depend on internal learning processes and competence transformation mechanisms. Yet the existing literature has not sufficiently explained whether digital training needs are directly associated with rural teachers’ cognitive learning ability and teaching ability, through what mechanism such associations may occur, or under what conditions these relationships may vary. This gap is particularly important in rural contexts, where teachers often face heavy workloads, limited time, and constrained access to high-quality digital learning resources. Accordingly, this study examines whether digital literacy mediates the relationship between digital training needs and two ability outcomes, and whether learning engagement serves as a boundary condition in this process.

### 1.1 Digital training needs and rural teachers’ cognitive learning ability and teaching ability (H1, H2)

Digital training needs refer to the motivations and goal orientations that rural teachers develop in response to the demands of educational digital transformation, aiming to promote their professional development and enhance their competencies (Xu, 2022). Cognitive learning ability is defined as the capacity to understand, organize, apply, analyze, integrate, and evaluate learning content, as well as to regulate one’s learning process effectively (Bloom, 1984; Seufert et al., 2024; Zimmerman, 2002). In the present study, this construct refers to the cognitive processing of training content rather than teachers’ technical ability to use digital tools. As digital training needs increase, teachers are more likely to actively seek learning opportunities relevant to digital teaching and devote greater effort to understanding and processing training content, thereby accumulating richer learning experiences (Gu et al., 2025). In this process, teachers must not only filter, understand, and process information, but also integrate new knowledge and reflect on the learning outcomes (Novoa-Echaurren et al., 2025). Consequently, learning activities evolve from a lower-order exposure—operation phase to a higher-order cognitive process that involves understanding—integration—transfer. Thus, digital training needs are not merely expressed as a willingness to participate in training, but also, through enhancing learning behaviors and cognitive engagement, may facilitate the improvement of cognitive learning abilities in areas such as understanding, analysis, integration, and evaluation (You et al., 2025). Based on this, Hypothesis 1 is proposed: Digital training needs are significantly positively related to rural teachers’ cognitive learning abilities; higher digital training needs are likely associated with stronger cognitive learning abilities.

Teaching ability, in this study, refers to the practical ability of teachers to effectively translate learned digital knowledge and tools into teaching design, classroom implementation, and learning support (Darling-Hammond et al., 2017). The stronger the digital training needs, the more likely teachers are to actively acquire and experiment with new digital resources and tools, integrating them into lesson planning, classroom organization, interactive feedback, and assessment, thereby promoting the updating of teaching strategies and the optimization of instructional processes (Blundell et al., 2016). Particularly in rural schools with relatively limited resources, digital tools and platforms often help expand the boundaries of teaching resources and improve teaching support efficiency, making “demand-driven learning” more likely to translate into “practical teaching improvements” (Karacaoğlu et al., 2025). Thus, digital training needs not only reflect the urgency for enhancing digital competencies, but may also deepen the classroom application of digital tools, further enhancing teaching ability (Wijnen et al., 2023). Based on this, Hypothesis 2 is proposed: Digital training needs are significantly positively related to rural teachers’ teaching abilities; higher digital training needs are likely associated with stronger teaching abilities.

### 1.2 Digital training needs → digital literacy → cognitive learning ability and teaching ability (H3, H4)

Digital literacy refers to an individual’s ability to effectively acquire, understand, evaluate, and create information in a digital environment, and to proficiently, critically, and responsibly use digital technologies for learning, communication, collaboration, and problem-solving (Gilster, 1997; Taheri and Pennington, 2024). Unlike cognitive learning ability, digital literacy emphasizes competence in using and evaluating digital tools and resources in digital contexts. The higher the level of digital training needs, the more likely teachers are to actively participate in digital training, consistently engage with digital resources and tools, and gain more frequent hands-on experience and problem-solving skills throughout the learning process, thereby promoting the development of their digital literacy (Luo et al., 2025). Digital literacy not only includes basic technical skills but also encompasses the ability to search, filter, and evaluate digital information, as well as the capability to engage in learning and communication collaboration in a digital environment (Liu and Xu, 2025). As digital literacy improves, teachers are more likely to effectively access, evaluate, and use digital information and tools in training contexts, which in turn creates more favorable conditions for understanding training materials, integrating knowledge, and transferring what they have learned. In this way, digital literacy may support the development of cognitive learning ability in areas such as comprehension, analysis, integration, and evaluation (Nagel and Amdam, 2025). Based on this, Hypothesis 3 is proposed: Digital literacy may play a significant mediating role between digital training needs and rural teachers’ cognitive learning ability, meaning that higher digital training needs are likely to lead to higher digital literacy, which in turn strengthens cognitive learning ability.

In rural schools, where digital resources and professional support are relatively scarce, the demand for digital training often encourages teachers to actively engage in training and online learning, allowing them to more frequently use digital tools in lesson preparation, resource retrieval, and classroom organization. As a result, the continuous learning and practical experiences accumulate into a relatively stable level of digital literacy (Luo et al., 2025). The enhancement of digital literacy further provides a key “transformation condition” for the development of teaching ability, enabling teachers to more effectively select, integrate, and apply digital resources. This allows for the embedding of technology in instructional design and classroom implementation, improving classroom interaction and the quality of learning support, and using tools and data to conduct process evaluations and reflective teaching. Consequently, this leads to a systemic improvement in teaching competence (Heine et al., 2023). Based on this, Hypothesis 4 is proposed: The relationship between digital training needs and teaching ability is more likely to be indirectly achieved through digital literacy as a mediating variable.

### 1.3 Digital training needs → digital literacy: moderating prediction of learning engagement (H5)

Learning engagement is typically viewed as the time, effort, and cognitive resources that learners invest in learning activities (Fredricks et al., 2004). It is generally associated with better learning continuity and quality. However, in the context of digital training for rural teachers, the influence of learning engagement on the pathway of “digital training needs → digital literacy” may not necessarily follow a simple linear enhancement. From the perspective of conservation of resources theory, individuals’ time, energy, and attention are limited resources, and excessive investment under constrained conditions may reduce the efficiency of competence development (Hobfoll, 1989). In addition, cognitive load theory suggests that when learners face sustained demands, high levels of effort may also be accompanied by overload, thereby weakening the extent to which training needs are translated into stable competence gains (Sweller, 1988). Rural teachers often face practical constraints such as limited resources, heavy teaching workloads, and fragmented time. Excessive learning engagement may lead to cognitive overload, mental exhaustion, and financial strain, resulting in a marginal effect of “investment → stress → reduced efficiency,” making it difficult for teachers to internalize demand-driven learning activities into a stable digital competence structure (Zhiyong and Hong, 2024). Additionally, in high-engagement scenarios, teachers may develop digital competence through self-directed learning, repeated practice, and peer exchange, which may weaken the extent to which perceived training needs alone predict improvements in digital literacy (Perera et al., 2019). Therefore, this study considers learning engagement as a key boundary condition and examines its moderating effect on the relationship between digital training needs and digital literacy. Based on this, Hypothesis 5 is proposed: Learning engagement negatively moderates the relationship between digital training needs and digital literacy, meaning that the higher the level of learning engagement, the weaker the positive association between digital training needs and digital literacy.

### 1.4 Research model and hypotheses

Based on the previous theoretical analysis, two models of “Digital Training Needs → Digital Literacy → Capability Enhancement” are proposed, focusing on the ability development path of rural teachers in the context of educational digitalization. The model takes digital training needs as the independent variable, cognitive learning ability and teaching ability as the dependent variables, and digital literacy as the key mediating variable, explaining how digital training needs translate into improvements in teachers’ abilities. Considering the practical constraints of rural teachers in terms of economic, time, and task pressures, learning engagement is introduced as a boundary condition to examine its moderating effect on the path from digital training needs to digital literacy.

**FIGURE 1:**
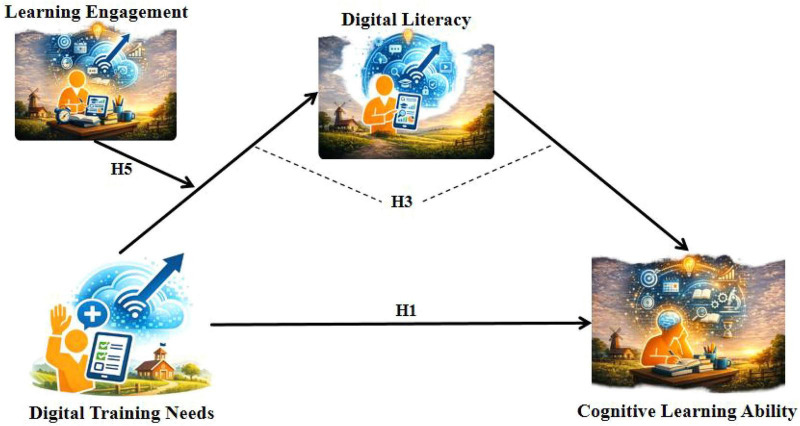
Moderated mediation model 1 (dependent variable: cognitive learning ability).

**FIGURE 2:**
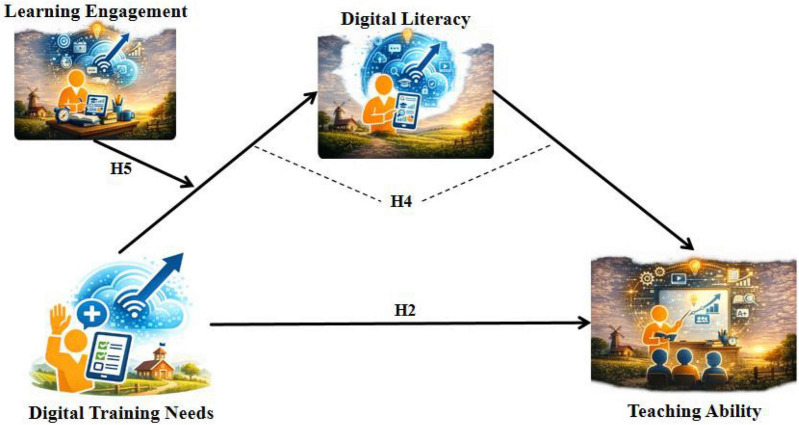
Moderated mediation model 2 (dependent variable: teaching ability).

The research hypotheses and model diagrams (Figures 1, 2) are as follows:

*H1*: Digital training needs have a significant positive association with rural teachers’ cognitive learning ability (Figure 1, H1).

*H2*: Digital training needs have a significant positive association with rural teachers’ teaching ability (Figure 2, H2).

*H3*: Digital literacy has a positive mediating effect between digital training needs and rural teachers’ cognitive learning ability (Figure 1, H3).

*H4*: Digital literacy has a positive mediating effect between digital training needs and rural teachers’ teaching ability (Figure 2, H4).

*H5*: Learning engagement negatively moderates the relationship between digital training needs and digital literacy, such that the higher the level of learning engagement, the weaker the positive association between digital training needs and digital literacy (Figures 1, 2, H5).

## 2 Materials and methods

### 2.1 Participants

An online questionnaire survey was conducted, targeting in-service teachers from elementary, middle, and high schools in rural areas of northern China. Based on the feasibility of the study, convenience sampling was used (Golzar et al., 2022), and the questionnaire was distributed through the network of schools and teachers accessible to the research team. This sampling strategy was adopted because no complete sampling frame of rural teachers across the target areas was available, and access to schools depended on administrative coordination and voluntary participation. Under these field constraints, convenience sampling was considered a practical approach to reach in-service rural teachers from multiple school levels and subject areas. Therefore, the sample is limited in terms of geographic region and context. To enhance the coverage and heterogeneity, the sample included teachers from various subjects, including Chinese, mathematics, English, science (physics, chemistry, biology), and humanities (moral education, history, geography, etc.). The explanatory boundaries of the findings are primarily based on rural teacher groups similar to the sample, and caution is advised when generalizing the results to other regions or contexts.

Given that the target population of the study is rural teachers, a pre-screening question was included in the sample selection. The questionnaire first asked whether the respondent was teaching in a rural area; if the answer was “no,” the system automatically terminated the response to exclude urban teachers. Before administering the questionnaire, participants were informed that the study adhered to principles of anonymity and confidentiality, participation was entirely voluntary, and they could withdraw at any point without facing any negative consequences.

**FIGURE 3:**
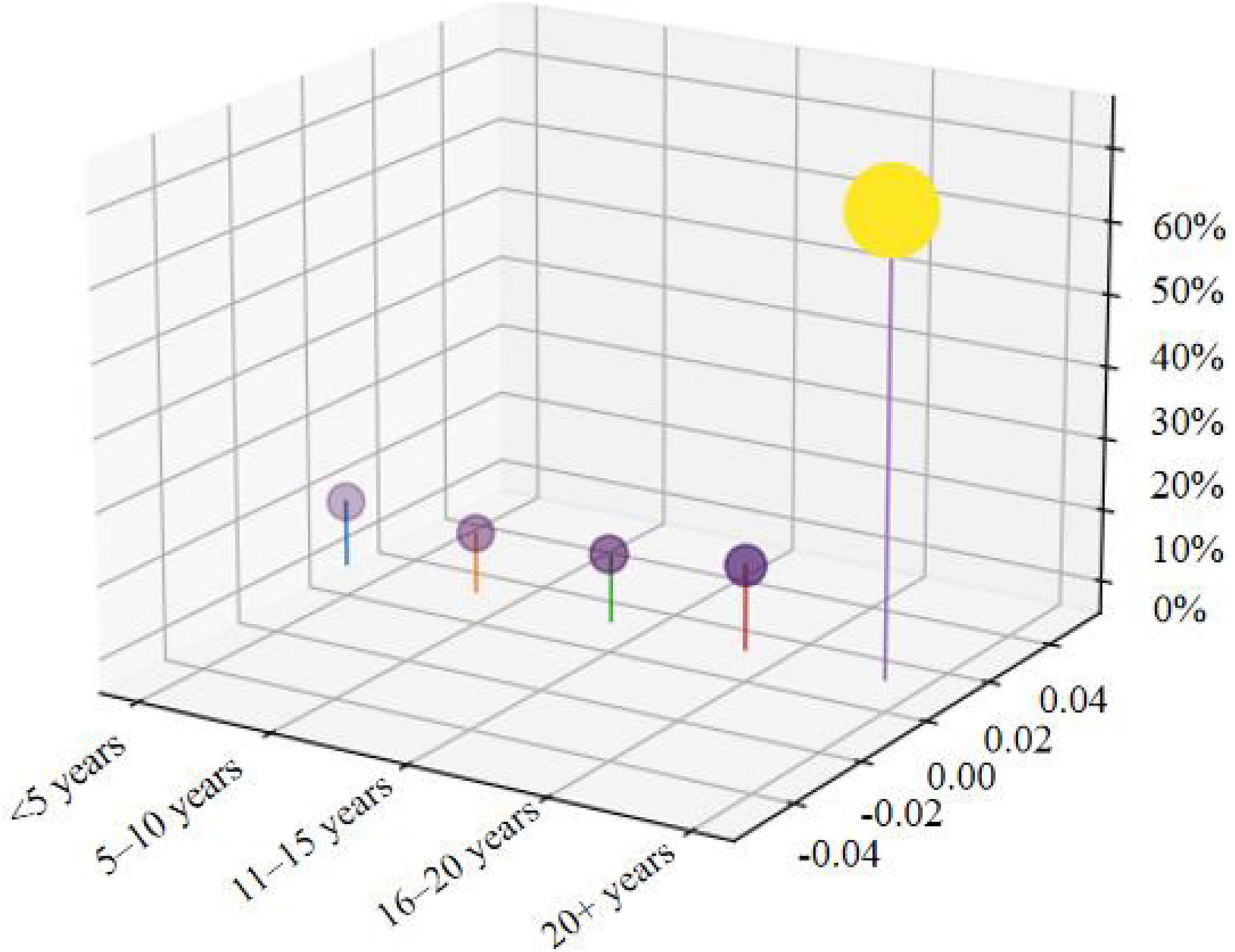
Distribution of teaching experience (*N* = 844).

A total of 919 rural teachers from China were recruited. After excluding 75 invalid questionnaires based on completeness and response consistency criteria, 844 valid samples were obtained, resulting in an effective response rate of 91.83%. This sample size was considered adequate in light of common recommendations for factor analysis and structural modeling, particularly because the study involved 24 observed items and retained two independent subsamples of 422 cases for EFA and CFA, respectively. The sample consisted of 450 males (53.32%) and 394 females (46.68%). The distribution of teaching experience is shown in Figure 3.

### 2.2 Measurement tools

To balance theoretical rigor with contextual relevance for rural teachers in mainland China, this study adapted existing questionnaires based on the theoretical foundations of prior research on digital literacy, teacher professional competence, and educational technology adoption. The adaptation process was iteratively revised using interview data, policy documents, and empirical studies related to digital training for rural teachers. The scale development followed the standard process for constructing psychological and educational measurement tools, including literature review, conceptual definition, expert review, exploratory and confirmatory factor analysis, pre-testing, and continuous revisions (Artino et al., 2014).

First, the study systematically reviewed the literature and existing measurement tools related to digital training needs, digital literacy, cognitive learning ability, and teaching ability, forming an initial conceptual framework and item pool. Second, to enhance content relevance and ecological validity, semi-structured interviews were conducted with a group of rural teachers. The teachers were first guided to describe their experiences, difficulties, and needs in digital learning and training. Then, focus questions were used to test the consistency of their understanding with the theoretical conceptual definitions. The interview results were used to refine conceptual boundaries and supplement contextual expressions and item content.

Third, based on the theoretical framework and interview results, a draft questionnaire was created and evaluated for content validity by six experts in the fields of educational technology, rural education, and teacher development. Experts provided revision suggestions from the dimensions of conceptual clarity, wording accuracy, contextual relevance, and structural rationality, and the research team adjusted item wording and structure accordingly.

Fourth, EFA and CFA were used to test the scale structure. The KMO and Bartlett’s test of sphericity prior to the EFA indicated that the data were suitable for factor analysis. Factor structures were then extracted, and items that did not align with theoretical expectations were adjusted. The CFA results showed that the model fit met common criteria (χ^2^*/df*, GFI, AGFI, TLI, CFI, RMSEA), and the standardized factor loadings were significant. The CR and AVE were within acceptable ranges, and the discriminant validity tests supported conceptual differentiation.

Finally, a pre-test was conducted with 30 rural teachers, and further adjustments were made to item wording, contextual expressions, and operational clarity based on their responses and feedback, resulting in the final version of the questionnaire. The scale used a 5-point Likert scale, ranging from 1 (strongly disagree) to 5 (strongly agree), with higher scores indicating higher levels. The specific items for each variable and their Cronbach’s α reliability coefficients are shown in Table 1.

**TABLE 1:** Measurement model construction reliability.

Variable	Theoretical basis	Items	CITC	α if deleted	α
Digital training needs	(Gómez-Trigueros et al., 2024)	I participate in digital training for job promotion.	0.670	0.830	0.844
		I participate in digital training to expand my professional knowledge.	0.717	0.778	
		I participate in digital training to improve my professional competence.	0.753	0.742	
Learning engagement	(Li and Yu, 2015; Fredricks et al., 2004)	I can apply effective learning strategies in digital training.	0.792	0.896	0.917
		I can monitor and regulate myself effectively in digital training.	0.817	0.892	
		I can be focused and persistent in digital training.	0.799	0.895	
		I can appreciate the value of learning in digital training.	0.791	0.897	
		I am interested in digital training.	0.733	0.909	
Digital literacy	(Peng and Zhu, 2024)	I have the consciousness of actively acquiring digital information and can actively explore and learn knowledge.	0.797	0.863	0.898
		I have acquired rich digital knowledge, such as the theory of digital technology and the history of technological development.	0.792	0.862	
		I have the ability to actively explore and acquire digital information.	0.788	0.863	
		I can strictly abide by digital ethics, such as being civilized online and not spreading false information on the Internet.	0.726	0.886	
Cognitive learning ability	(Bloom, 1984)	I can memorize digital training content proficiently.	0.749	0.910	0.921
		I can understand digital training content well.	0.769	0.907	
		I can apply digital training content well.	0.761	0.908	
		I can analyze the relationship between components of digital training content.	0.787	0.905	
		I can integrate components of digital training content.	0.781	0.906	
		I can accurately assess the value of digital training content.	0.798	0.903	
Teaching ability	(Zhang and Huang, 2023)	I can abide by the code of ethics and practice the ethics of teachers.	0.812	0.926	0.937
		I can teach students a wide range of subject knowledge.	0.802	0.927	
		I can make a good teaching plan.	0.802	0.927	
		I can carry out good curriculum teaching activities.	0.838	0.923	
		I can accurately assess the learning effectiveness of students.	0.822	0.925	
		I can reflect on the shortcomings in teaching in a timely manner.	0.805	0.927	

References indicate the theoretical grounding of each construct rather than the source of the specific items; CITC, corrected item–total correlation; α if deleted, Cronbach’s alpha if the item is deleted; α, Cronbach’s alpha.

### 2.3 Data analysis

The main statistical analysis of this study aims to test two moderated mediation models, using SPSS 26.0 and its PROCESS macro. First, Cronbach’s α coefficients for the five scales are calculated to assess internal consistency reliability, followed by EFA. Next, CFA is performed, and CMB is tested. In addition to statistical testing, several procedural remedies were adopted to reduce common method bias, including anonymous data collection, voluntary participation, confidentiality assurance, and clear instructions to encourage honest responses. Descriptive statistics and correlation analysis are then conducted to examine the relationships between variables. Finally, PROCESS (Model 7) is used to test the mediating role of digital literacy and the moderating prediction of learning engagement in the relationship between digital training needs and cognitive learning ability/teaching ability. The significance of indirect effects was further examined using bootstrap confidence intervals based on 5,000 resamples. Simple slope plots of the moderation effects are further drawn to assist in interpreting the interaction effects. Standardized coefficients are used for model testing, with the significance level set at *p* < .05. Because the data were collected at a single time point, the analyses were designed to identify associations and indirect statistical pathways rather than to support causal inference.

The total regression equation for the dependent variable, cognitive learning ability (CLA), is as follows:


$$C\,L\,A=\beta_{0}+\beta_{1}\,D\,T\,N+\beta_{2}\,D\,L+\beta_{3}\,\left(D\,T\,N\times L\,E\right)+\varepsilon$$

The total regression equation for the dependent variable, teaching ability (TA), is as follows:


$$T\,A=\gamma_{0}+\gamma_{1}\,D\,T\,N+\gamma_{2}\,D\,L+\gamma_{3}\,\left(D\,T\,N\times L\,E\right)+\varepsilon$$

Where β_0_ or γ_0_ represents the constant term; β_1_ DTN or γ_1_ DTN represents the main effect of digital training needs (DTN); β_2_ DL or γ_2_ DL represents the mediating effect of digital literacy (DL); and β_3_ (DTN × LE) or γ_3_ (DTN × LE) represents the interaction term between digital training needs (DTN) and learning engagement (LE).

## 3 Results

### 3.1 Exploratory factor analysis

The first 50% of the sample (422 responses) was selected as an independent dataset for factor extraction. The results showed that the eigenvalues of the first five factors were all greater than 1, and the scree plot displayed a clear inflection point after the fifth factor. According to the “eigenvalue > 1” criterion, five common factors were retained (Wang et al., 2025). The initial variance contribution rates of the five factors were 15.321, 8.976, 4.264, 2.445, and 2.173%, respectively. The cumulative variance explained was 69.858%, which is higher than the recommended threshold of 60% (Sellbom and Goretzko, 2023), indicating that the extracted factors effectively explain most of the variance in the original variables. After rotation, the variance contribution rates of the five factors were 11.328, 11.207, 10.575, 10.461, and 9.758%, showing a relatively balanced distribution of explanatory power across the factors.

The rotated factor loading matrix indicated that the factor loadings for digital training needs (three items) were 0.797, 0.827, and 0.893, effectively reflecting rural teachers’ need for digital training. The factor loadings for learning engagement (five items) were 0.759, 0.769, 0.751, 0.780, and 0.803, reflecting the participants’ level of engagement in learning. The factor loadings for digital literacy (four items) were 0.752, 0.816, 0.802, and 0.778, representing individuals’ level of digital literacy. The rotated factor loadings for cognitive learning ability (six items) were 0.724, 0.791, 0.733, 0.799, 0.749, and 0.761, reflecting individuals’ cognitive learning abilities. The factor loadings for teaching ability (six items) were 0.731, 0.830, 0.724, 0.755, 0.763, and 0.787, showing significant explanatory power for individuals’ teaching performance. Overall, the five-factor structure extracted is highly consistent with the proposed theoretical model, demonstrating strong theoretical validity and statistical support.

### 3.2 Confirmatory factor analysis

The remaining 50% of the sample (422 responses) was used as an independent dataset for CFA. As shown in Table 2, the standardized factor loadings of all observed variables ranged from 0.717 to 0.865, and all were statistically significant (*p* < 0.001), indicating a strong correlation between each observed variable and its corresponding latent variable. Additionally, the CR values for the latent variables ranged from 0.832 to 0.936, and the AVE values ranged from 0.623 to 0.709, demonstrating good internal consistency and convergent validity of the measurement tool. The overall model met the criteria for evaluation, with standardized factor loadings > 0.50, CR > 0.70, and AVE > 0.50 (Fornell and Larcker, 1981). Therefore, the constructs demonstrated good reliability and convergent validity.

**TABLE 2:** Summary of CFA results, composite reliability, and average variance extracted.

Variable	Items	Estimate	*S.E.*	*Z*	*p*	*Std.*	SMC	CR	AVE
Digital training needs	Q1	1	0.062	15.142	***	0.717	0.514	0.832	0.623
	Q2	0.933				0.808	0.653		
	Q3	1.006	0.065	15.568	***	0.838	0.702		
Learning engagement	Q4	1	0.056	18.835	***	0.784	0.615	0.910	0.670
	Q5	1.059				0.843	0.711		
	Q6	1.120	0.059	18.969	***	0.848	0.719		
	Q7	1.116	0.058	19.134	***	0.854	0.729		
	Q8	1.042	0.063	16.544	***	0.759	0.576		
Digital literacy	Q9	1	0.057	18.992	***	0.818	0.669	0.890	0.671
	Q10	1.074				0.830	0.689		
	Q11	1.100	0.055	19.870	***	0.864	0.746		
	Q12	0.946	0.056	16.955	***	0.760	0.578		
Cognitive learning ability	Q13	1	0.059	18.154	***	0.798	0.637	0.921	0.660
	Q14	1.068				0.798	0.637		
	Q15	1.044	0.058	17.857	***	0.788	0.621		
	Q16	1.013	0.055	18.481	***	0.809	0.654		
	Q17	1.040	0.055	18.920	***	0.823	0.677		
	Q18	1.113	0.056	19.897	***	0.855	0.731		
Teaching ability	Q19	1	0.043	21.260	***	0.858	0.736	0.936	0.709
	Q20	0.919				0.820	0.672		
	Q21	0.977	0.043	22.549	***	0.848	0.719		
	Q22	0.945	0.040	23.352	***	0.865	0.748		
	Q23	0.938	0.043	22.006	***	0.837	0.701		
	Q24	0.989	0.046	21.437	***	0.824	0.679		

*n* = 422. ***p < 0.001.

### 3.3 Common method bias test

To examine whether common method bias existed in this study, both procedural and statistical remedies were considered (Podsakoff et al., 2003). Procedurally, the questionnaire was administered anonymously, participation was voluntary, confidentiality was emphasized, and respondents were informed that there were no right or wrong answers, which helped reduce evaluation apprehension and response distortion. Statistically, a single-factor confirmatory factor analysis was conducted using AMOS. All measurement variables were loaded onto a single latent factor to construct the single-factor model, and the moderated mediation model was first tested with cognitive learning ability as the dependent variable. The results indicated poor model fit: χ^2^ = 784.610, *df* = 135, χ^2^*/df* = 5.812, GFI = 0.790, AGFI = 0.734, TLI = 0.878, CFI = 0.893, RMSEA = 0.107, all of which failed to meet the model fit criteria proposed by Hu and Bentler (1999) (χ^2^*/df* ≤ 3; GFI, AGFI, TLI, and CFI ≥ 0.90; RMSEA ≤ 0.08). This suggests that a single factor could not adequately account for the covariance among all measurement variables. Subsequently, a full measurement model including all latent variables was tested, and the results demonstrated good model fit: χ^2^ = 81.360, *df* = 72, χ^2^*/df* = 1.130, GFI = 0.951, AGFI = 0.942, TLI = 0.997, CFI = 0.997, RMSEA = 0.015. The moderated mediation model with cognitive learning ability as the dependent variable also showed satisfactory fit, and therefore is not elaborated further here. In sum, there was no serious common method bias in this study.

### 3.4 Descriptive statistics and correlation analysis

Although the means of the variables are relatively high, their distributions do not show a clear tendency to concentrate at the upper limit, indicating that potential ceiling effects and range restrictions are unlikely to cause substantial bias in the correlations. As shown in Table 3, the means (*M*) of the variables range from 3.46 to 4.41, all falling within a relatively positive evaluation range, rather than being highly concentrated near the highest scores. The absolute values of skewness range from 1.33 to 1.56 ( < 2), and the absolute values of kurtosis range from 1.80 to 2.71 ( < 7), meeting the conventional criteria for approximate normal distribution (George, 2011). Furthermore, AVE was used to test the discriminant validity among the five variables. The square roots of the AVEs for all variables are greater than the standardized correlation coefficients between the corresponding constructs, indicating good discriminant validity (Miao and Zhang, 2024). As shown in Table 3, all focal variables were positively and significantly correlated, with especially strong associations of digital literacy with teaching ability (*r* = 0.891, *p* < 0.001) and cognitive learning ability (*r* = 0.798, *p* < 0.001), which provides preliminary support for the subsequently tested mediation models.

**TABLE 3:** Descriptive statistics, normality indices, and inter-construct correlations.

Variable	*M*	*SD*	Skew	Kurtosis	1	2	3	4	5
1. Digital training needs	4.33	0.76	−1.34	1.84	***0.789***	***0.819***	***0.819***	***0.812***	***0.842***
2. Learning engagement	3.46	0.78	−1.33	1.80	0.705***				
3. Digital literacy	4.40	0.72	−1.56	2.97	0.803***	0.741***			
4. Cognitive learning ability	4.29	0.74	−1.33	2.04	0.784***	0.845***	0.798***		
5. Teaching ability	4.41	0.71	−1.55	2.71	0.784***	0.732***	0.891***	0.789***	

*N* = 844. *p < 0.05, **p < 0.01, ***p < 0.001. All tests were two-tailed; Values in bold italics represent the square root of the AVE.

### 3.5 Moderated mediation model 1 test (dependent variable: cognitive learning ability)

**FIGURE 4:**
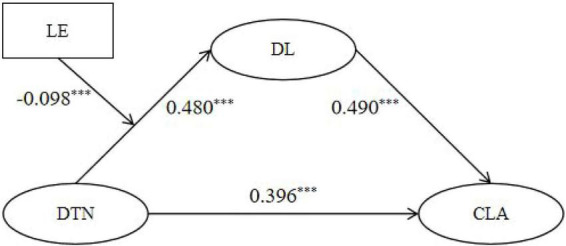
Path coefficients of moderated mediation model 1. DTN, digital training needs; LE, learning engagement; DL, digital literacy; CLA, cognitive learning ability. *p < 0.05, **p < 0.01, ***p < 0.001.

As shown in Table 4 and Figure 4, the final dependent variable is cognitive learning ability (CLA). The mediation relationship of digital training needs (DTN) → digital literacy (DL) → cognitive learning ability (CLA) was tested, and the moderating effect of learning engagement (LE) on the DTN → DL relationship was examined.

**TABLE 4:** Moderated mediation model 1 results.

Name/Variable		DV: DL				DV: CLA			
		β	S.E.	*t*	95% CI	β	S.E.	*t*	95% CI
IV	DTN	0.480***	0.259	18.531	[0.429, 0.531]	0.396***	0.031	12.628	[0.335, 0.458]
MO	LE	0.287***	0.243	11.812	[0.240, 0.335]	–	–	–	–
ME	DL	–	–	–	–	0.490***	0.033	14.846	[0.426, 0.555]
IT	DTN × LE	−0.098***	0.016	−6.114	[−0.130, −0.067]	−	−	−	−
*R*		0.847				0.834			
*R* ^2^		0.717				0.695			
*F(df1, df2)*		710.794***(3, 840)				956.670***(2, 841)			

***p < 0.001. IV, Independent; MO, Moderating; ME, Mediating; IT, Interaction Term; DV, Dependent Variable; DTN, Digital Training Needs; LE, Learning Engagement; DL, Digital Literacy; CLA, Cognitive Learning Ability.

In the results equation (DV = CLA), the direct relationship between digital training needs and cognitive learning ability was significantly positive [β = 0.396, *t* = 12.628, 95% CI (0.335, 0.458), supporting H1]. This coefficient indicates a moderately strong positive association, suggesting that teachers with higher digital training needs tend to report meaningfully better cognitive learning ability. Both the relationship between digital training needs and digital literacy and that between digital literacy and cognitive learning ability were significantly positive [β = 0.480, *t* = 18.531, 95% CI (0.429, 0.531); β = 0.490, *t* = 14.846, 95% CI (0.426, 0.555), supporting H3], confirming the mediation effect of digital literacy. In addition, the indirect effect of digital training needs on cognitive learning ability via digital literacy was significant based on bootstrap estimation, because the 95% bootstrap confidence interval did not include zero. This indicates that part of the association between digital training needs and cognitive learning ability operates through teachers’ digital literacy.

In the mediation equation (DV = DL), learning engagement showed a significantly positive relationship with digital literacy [β = 0.287, *t* = 11.812, 95% CI (0.240, 0.335)]. The interaction term DTN × LE had a significantly negative relationship with digital literacy [β = −0.098, *t* = −6.114, 95% CI (-0.130, −0.067), supporting H5].

In summary, there is a direct relationship between digital training needs and cognitive learning ability, with digital literacy playing a mediating role. Learning engagement negatively moderates the path of “digital training needs → digital literacy,” meaning that the higher the level of learning engagement, the weaker the positive relationship between digital training needs and digital literacy. In practical terms, these coefficients suggest that rural teachers’ perceived digital training needs are not merely associated with stronger motivation to participate in training, but are also meaningfully linked to better cognitive processing of training content, especially through the enhancement of digital literacy. This indicates that need-aligned training may help teachers not only attend training, but also better understand, integrate, and apply what they learn.

### 3.6 Moderated mediation model 2 test (dependent variable: teaching ability)

**FIGURE 5:**
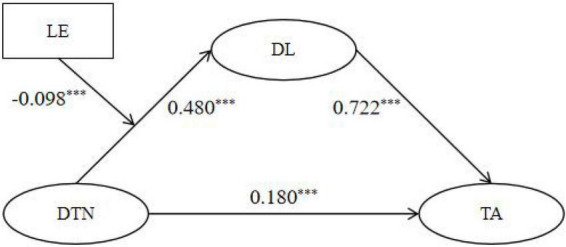
Path coefficients of moderated mediation model 2. DTN, digital training needs; LE, learning engagement; DL, digital literacy; TA, teaching ability. *p < 0.05, **p < 0.01, ***p < 0.001.

As shown in Table 5 and Figure 5, with teaching ability (TA) as the final dependent variable, the mediation effect of digital training needs (DTN) → digital literacy (DL) → teaching ability (TA) was tested, and the moderating effect of learning engagement (LE) on the DTN → DL relationship was examined.

**TABLE 5:** Moderated mediation model 2 results.

Name/Variable		DV: DL				DV: TA			
		β	S.E.	*t*	95% CI	β	S.E.	*t*	95% CI
IV	DTN	0.480***	0.259	18.531	[0.429, 0.531]	0.180***	0.023	7.599	[0.133, 0.226]
MO	LE	0.287***	0.243	11.812	[0.240, 0.335]	–	–	–	–
ME	DL	–	–	–	–	0.722***	0.025	29.020	[0.674, 0.771]
IT	DTN × LE	−0.098***	0.016	−6.114	[−0.130, −0.067]	–	–	–	–
*R*		0.847				0.899			
*R* ^2^		0.717				0.807			
*F(df1, df2)*		710.794***(3, 840)				1761.876***(2, 841)			

***p < 0.001. IV, Independent; MO, Moderating; ME, Mediating; IT, Interaction Term; DV, Dependent Variable; DTN, Digital Training Needs; LE, Learning Engagement; DL, Digital Literacy; TA, Teaching Ability; DTN, Digital Training Needs; LE, Learning Engagement; DL, Digital Literacy; TA, Teaching Ability.

In the results equation (DV = TA), the direct relationship between digital training needs and teaching ability was significantly positive [β = 0.180, *t* = 7.599, 95% CI (0.133, 0.226), supporting H2]. Although this direct effect is smaller than that observed for cognitive learning ability, it still indicates a meaningful positive association, suggesting that stronger digital training needs are linked to better teaching-related performance. The relationships between digital training needs and digital literacy, and between digital literacy and teaching ability, were also significantly positive [β = 0.480, *t* = 18.531, 95% CI (0.429, 0.531); β = 0.722, *t* = 29.020, 95% CI (0.674, 0.771), supporting H4], confirming the mediation effect of digital literacy. In addition, the indirect effect of digital training needs on teaching ability via digital literacy was significant based on bootstrap estimation, because the 95% bootstrap confidence interval did not include zero. This suggests that digital literacy is an important pathway through which teachers’ digital training needs are linked to teaching ability.

In the mediation equation (DV = DL), learning engagement showed a significantly positive relationship with digital literacy [β = 0.287, *t* = 11.812, 95% CI (0.240, 0.335)]. The interaction term DTN × LE had a significantly negative relationship with digital literacy [β = −0.098, *t* = −6.114, 95% CI (-0.130, −0.067), supporting H5].

In summary, the direct relationship between digital training needs and teaching ability is supported, and the mediation effect of digital literacy is confirmed. The moderating effect of learning engagement on the path “digital training needs → digital literacy” is further validated, showing a significant negative prediction: the higher the level of learning engagement, the weaker the positive predictive effect of digital training needs on digital literacy. Practically, this pattern suggests that when rural teachers perceive stronger digital training needs, they are more likely to translate those needs into improvements in teaching-related competence, particularly when those needs are accompanied by stronger digital literacy. In other words, digital literacy appears to function as an important bridge through which training needs are connected with classroom-related capability improvement.

### 3.7 Moderation effect slope test

**FIGURE 6:**
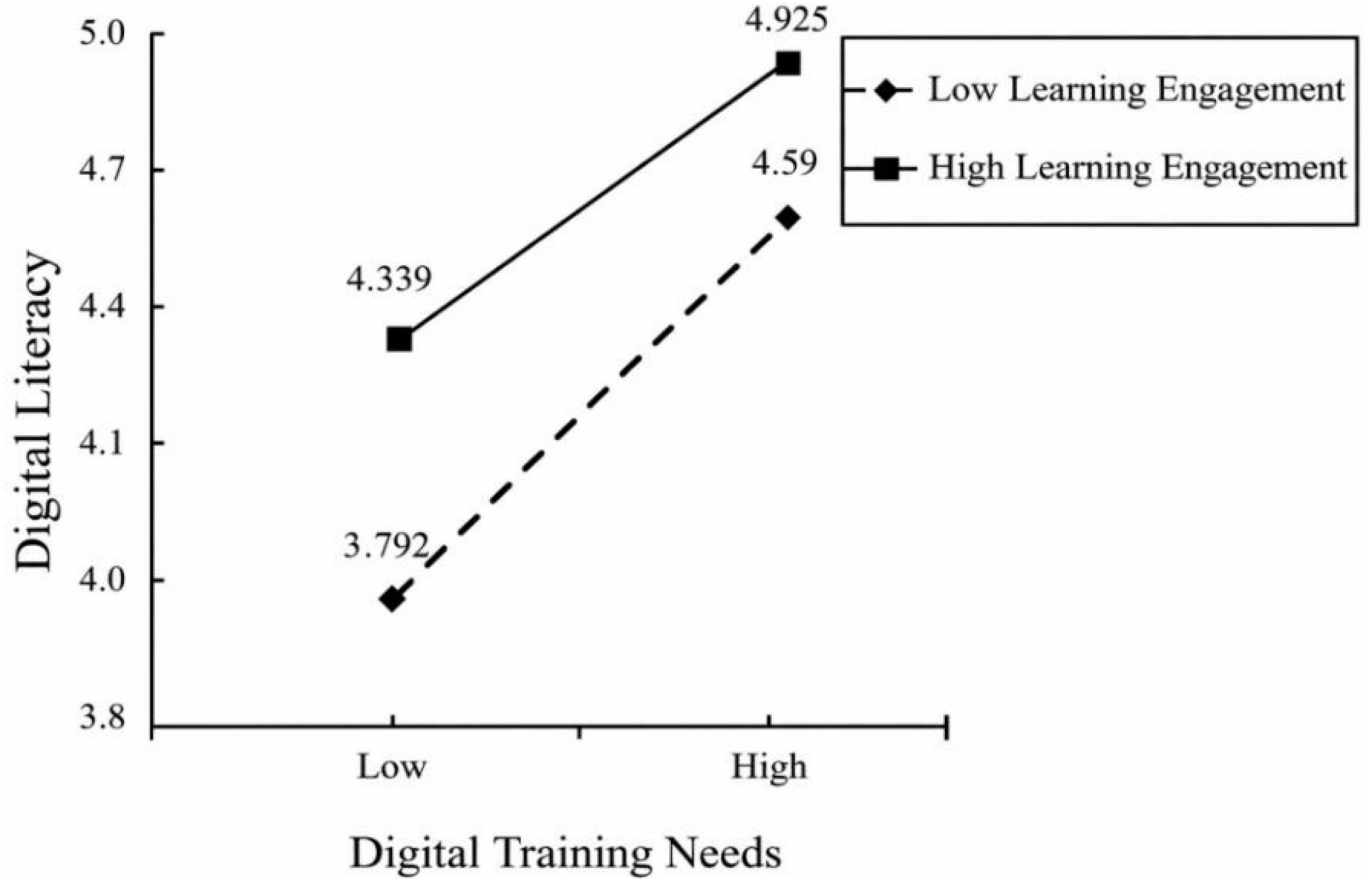
Slope visualization.

The slope visualization in Figure 6 shows that learning engagement has a significant moderating effect on the relationship between digital training needs and digital literacy. For teachers with low learning engagement, the path effect is stronger (β = 0.558, *t* = 22.314, *p* < 0.001). For teachers with high learning engagement, the path effect remains significant (β = 0.409, *t* = 12.963, *p* < 0.001), but the intensity is weaker. In conclusion, the predictive effect of digital training needs on digital literacy gradually weakens as the level of learning engagement increases, further supporting H5. As illustrated in Figure 6, the steeper slope for the low-learning-engagement group indicates that digital training needs are more strongly translated into digital literacy when teachers are relatively less engaged, whereas the flatter slope for the high-learning-engagement group indicates a weaker, though still positive, relationship.

## 4 Discussion

This study contributes to understanding how rural teachers’ digital training needs are linked with their learning- and teaching-related capabilities in the context of educational digital transformation. Rather than treating training needs as a simple indicator of demand, the findings suggest a broader “needs → literacy → ability” pathway, while also indicating that this pathway varies according to teachers’ level of learning engagement. The following discussion focuses on the theoretical meaning of these patterns, possible alternative explanations, and their implications for research and practice.

### 4.1 Digital training needs and rural teachers’ cognitive learning ability and teaching ability (supporting H1, H2)

The positive association between digital training needs and rural teachers’ cognitive learning ability suggests that perceived need may function as a motivational-cognitive trigger in teachers’ professional learning. When teachers perceive stronger digital training needs, they may become more aware of the gap between current competence and desired professional performance, which can activate more purposeful information processing, strategy use, and reflection during training (Trevisan et al., 2024). From a self-regulated learning perspective, such needs may help transform externally perceived pressure into internally organized learning goals, thereby strengthening monitoring, regulation, and deeper cognitive engagement with training content (Karlen and Hertel, 2024).

The positive association between digital training needs and teaching ability suggests that perceived developmental demands may also have practical implications for how teachers update and enact their instructional practice. Stronger digital training needs generally mean that teachers are more sensitized to the value of digital tools and resources for teaching organization, interactive support, and learning evaluation in the classroom context. They are also more likely to form ongoing tendencies for experimentation and integration (Amemasor et al., 2025). As a result, teachers are more likely to accumulate positive abilities in areas such as lesson preparation, resource acquisition, classroom activity design, teaching implementation, and feedback improvement (Gu et al., 2025). In theoretical terms, this pattern is consistent with the view that perceived training needs are not only diagnostic signals of skill gaps, but also orienting signals that may direct teachers’ effort toward experimentation, adaptation, and instructional improvement. Thus, digital training needs may matter not simply because they increase training participation, but because they help align professional learning with teachers’ perceived classroom demands.

### 4.2 Digital training needs → cognitive learning ability and teaching ability: mediating role of digital literacy (supporting H3, H4)

The mediating role of digital literacy suggests that digital training needs may be translated into capability development not automatically, but through a competence-conversion process. In this sense, digital literacy appears to function as a foundational enabling resource that helps transform perceived training needs into more effective learning and teaching performance.

This finding can be understood from the perspective of competency construction and resource transformation. Perceived training needs may increase teachers’ sensitivity to digital teaching demands, but such sensitivity is unlikely to improve outcomes unless it is converted into usable digital knowledge, judgment, and operational capacity (Yan et al., 2025). In this process, teachers are more likely to accumulate digital knowledge and operational strategies through training participation, peer collaboration, online resource use, and iterative classroom practices, thereby gradually improving their digital literacy (Shi et al., 2025). As a relatively foundational “general digital competency,” digital literacy supports teachers’ learning processes and teaching practices: at the cognitive level, it facilitates smoother information retrieval and evaluation, tool selection and strategy optimization, and learning monitoring and reflective regulation (Zhiyong and Hong, 2024); at the teaching level, it also makes it easier for teachers to integrate digital resources and tools into key aspects such as lesson preparation, classroom interaction, learning support, and feedback, aligning with higher levels of teaching ability (Zhang et al., 2024).

Because the study is based on cross-sectional data, the mediating pattern should be interpreted as an associative or predictive pathway rather than as direct evidence of causal sequencing. Overall, this finding highlights the pivotal role of digital literacy in the digital development of rural teachers and provides empirical evidence and theoretical insights for the subsequent discussion on how to optimize training provision and support systems to promote the development of teachers’ digital literacy, thereby enhancing both learning and teaching abilities.

### 4.3 Digital training needs → digital literacy: negative moderating effect of learning engagement (supporting H5)

The negative moderating effect of learning engagement suggests that the relationship between digital training needs and digital literacy is contingent rather than uniform across teachers.

This pattern may be understood through the lenses of diminishing marginal returns and resource substitution. Teachers with high learning engagement may already possess stronger self-regulated learning habits and multiple pathways for competence development, making their digital literacy less dependent on perceived training needs alone. Their digital literacy may come more from daily accumulation (such as self-exploration, repeated practice, peer collaboration, and online resource usage). Therefore, the strength of digital training needs has relatively little impact on differentiating their level of digital literacy (Lan et al., 2025). On the other hand, for teachers with low learning engagement, training needs serve more as an “external driving signal,” more clearly reflecting their ability gaps and learning motivation differences, which results in a stronger synchronous change with their digital literacy level (Lei et al., 2024). Second, high learning engagement means that teachers invest more time, energy, and attention to learning resources, and the path to acquiring digital literacy may be more diverse. The relative role of training needs is dispersed in this process (Cristea et al., 2025; Seufert, 2026). In contrast, when learning engagement is low, training needs are more likely to serve as an important clue to explain differences in digital literacy (Urhahne and Wijnia, 2023).

The above moderation results suggest that learning engagement is an important boundary condition in the “needs → literacy” linkage, indicating that training policies and school support should consider differences in teachers’ learning engagement when implemented: for those with low learning engagement, enhancing participation motivation, learning support, and process guidance may be more crucial; for those with high learning engagement, further provision of advanced and personalized digital competency development resources may be needed to match their ongoing learning and application deepening needs. In addition, the weaker association under high learning engagement may also reflect a ceiling or saturation tendency: once teachers are already highly engaged in learning, variation in perceived training needs may contribute less additional explanatory power to differences in digital literacy.

### 4.4 Implications for the broader educational context, including higher education

Although the present study focused on rural primary and secondary school teachers, the findings may also offer tentative implications for the broader educational context, including higher education. In particular, the pathway identified in this study—where digital training needs are associated with ability outcomes through digital literacy—suggests that, across educational sectors, teachers’ perceived training needs may function as an important developmental signal that supports competence growth when accompanied by sufficient digital literacy. In higher education, where instructors are also expected to integrate digital tools into teaching, course design, assessment, and student support (Novoa-Echaurren et al., 2025), the present findings imply that simply increasing training provision may be insufficient unless such training is aligned with teachers’ actual needs and contributes to the strengthening of digital literacy. At the same time, the moderating role of learning engagement suggests that the effectiveness of need-based digital development may vary across individuals and contexts. However, because higher education differs substantially from rural basic education in institutional conditions, professional roles, technological infrastructure, and instructional demands, these implications should be interpreted cautiously and require direct empirical verification in university settings.

### 4.5 Relation of the present findings to psychological literacy

Although psychological literacy was not directly assessed in this study, the present findings may still be discussed in relation to this concept. Psychological literacy is generally understood as the capacity to reflect on human behavior and mental processes and to apply psychological knowledge and skills in real-world personal, social, and professional contexts (McGovern et al., 2010; Cranney and Dunn, 2011). In this sense, there appears to be some conceptual overlap between psychological literacy and the constructs examined in the present study. Specifically, cognitive learning ability involves understanding, analysis, integration, evaluation, and reflective processing; learning engagement reflects sustained effort, self-regulation, and active participation in learning; and teaching ability concerns the translation of acquired knowledge and digital tools into instructional practice and learner support. These dimensions are broadly consistent with the view that educational development should extend beyond knowledge acquisition to include reflection, transfer, and application in authentic contexts (McGovern et al., 2010; Cranney and Dunn, 2011). At the same time, psychological literacy was not measured as a distinct construct in this study. Therefore, the connection between the current findings and psychological literacy should be interpreted as conceptual rather than empirical. Future research may further examine whether psychological literacy can serve as an additional explanatory construct linking teachers’ digital learning, engagement, and professional practice.

### 4.6 Implications in the age of AI

Although artificial intelligence was not directly examined in this study, the findings may still have implications for teacher development in the age of AI. As AI becomes increasingly embedded in educational practice, digital literacy needs to extend beyond the use of general digital tools to include critical understanding, pedagogical judgment, and ethical reflection regarding AI-supported technologies (UNESCO, 2023, 2024). In this sense, the present findings suggest that AI-related teacher development may be more effective when training is aligned with teachers’ actual needs and supports not only technical competence but also reflective and responsible use. In addition, the moderating role of learning engagement indicates that teachers may benefit differently from AI-related learning opportunities depending on their level of sustained participation and self-regulation. However, because AI-related competencies were not directly measured in this study, these implications should be interpreted cautiously and await further empirical verification. UNESCO’s recent AI framework for teachers defines teacher AI competence across five dimensions—human-centered mindset, ethics of AI, AI foundations and applications, AI pedagogy, and AI for professional learning—while its guidance on generative AI in education emphasizes human-centered, ethical, safe, and meaningful use, which further supports the relevance of extending the present discussion toward AI-era teacher development.

## 5 Limitations

First, this study uses cross-sectional questionnaire data, so the conclusions should be understood as associations and predictive relationships, rather than supporting causal inference or directional causal claims. The mediation and moderated mediation results should also not be interpreted as causal mechanisms. In addition, potential endogeneity cannot be ruled out, because reverse associations and unobserved teacher- or school-level factors may affect the estimated relationships. Future research could use longitudinal or quasi-experimental designs to strengthen temporal identification and reduce endogeneity concerns.

Second, the variables primarily come from self-reports by teachers, which may still be subject to common method bias and social desirability effects. Future studies could incorporate multi-source data (such as training platform behavior, classroom observations, manager/peer evaluations, etc.) to improve measurement objectivity.

Third, the sample is concentrated on rural teachers in North China, and the generalizability may be limited by regional and school-level differences in digital resources. It is recommended to conduct replication tests and cross-group comparisons in different regions and school types.

Fourth, key constructs have multidimensional attributes, and this study used overall scales, which may not fully capture dimensional differences. Future studies could conduct more fine-grained dimensional modeling or incorporate task-based assessments.

Fifth, the model does not include broader contextual and individual difference variables (e.g., organizational support, training quality, and digital self-efficacy). In addition, although the findings may be conceptually relevant to psychological literacy, this construct was not directly measured. Future research could examine these factors within a broader ecological framework and include psychological literacy as a distinct construct. In addition, although the findings may have implications for teacher development in the age of AI, AI-related competencies were not directly measured in the present study.

## 6 Conclusion

Based on a sample of rural teachers, this study examined the linkage of “digital training needs → digital literacy → ability.” The results show that digital training needs are positively associated with cognitive learning ability and teaching ability, that digital literacy serves as a key mediating pathway, and that learning engagement negatively moderates the relationship between digital training needs and digital literacy. The main contribution of this study is to move beyond a simple focus on training provision by identifying a moderated mediation mechanism through which teachers’ perceived digital training needs are linked to both learning- and teaching-related outcomes in a rural context.

These findings suggest that the digital development of rural teachers depends not only on the provision of training but also on teachers’ digital literacy and learning engagement. Practically, schools and training providers should first assess teachers’ actual digital training needs, then design tiered training content aligned with those needs, and embed digital literacy development into training tasks and follow-up support. In addition, teachers with lower learning engagement may need stronger participation guidance, feedback, and scaffolding, whereas those with higher learning engagement may benefit more from advanced, practice-oriented, and personalized digital learning opportunities.

## Data Availability

The raw data supporting the conclusions of this article will be made available by the authors, without undue reservation.
